# 

**Published:** 1886-06-20

**Authors:** 


### EDITORIAL NOTICES.

New Advertisements —Please examine the following new advertisements of the excellent establishments mentioned, to wit:

Tulaine University, Medical Department, New Orleans, La.

Southern Medical College, Atlanta, Ga.

Robinson's Hypophosphites and Robinson's Wine Coca.—We have examined samples of the above preparations. They are elegantly and neatly prepared, and the formulæ being published upon the labels will strike with favor any physician who desires to prescribe the drugs therein contained. The house of R. A. Robinson & Co., comes to us highly recommended as enterprising and reliable manufacturing chemists of Louisville, Ky. See their advertisement in this Journal.

The Christian Herald and Signs of Our Times.—This is the title of a most excellent Christian newspaper, well adapted for the ministers of the Gospel, for parents, and for family reading. It is a sixteen page folio, the pages about ten by twelve inches with three columns on a page, and each number is beautifully illustrated, every number containing well executed cuts often of prominent divines, and other distinguished persons in Europe and America. Each number contains a sermon from Spurgeon, and also one from Talmage with other varied and interesting matter. We commend the paper as one of great interest and usefulness. Terms $1 50. Address Christian Herald, 63 Bible House, New York City.

There Now.—The New York *Medical Times* (Homœopathic) says: Our Old School friends have this advantage over our New School friends, that no matter what their practice, in name at least they are non-sectarian, and they can gradually, as they are doing now, absorb the truths of all schools without eating any very large amount of crow. Our new school friends are thus placed at a disadvantage; however liberal they may be in practice, in name they (the New School) are certainly sectarian, and more or less restricted to a single line of therapeutics which closes the door against the minute scientific investigation of the principles which they assert are firmly established, and as unchangeable as the laws of the Medes and Persians."

RECEIPTED.

For 1886—R A. McQueen, A. F. Pharr, T. W. Loury, Jas. Jones, T. T. Spark, W. W. Pearce, Eli Smythe, L. Kiser, L. L. Hopewell, Robt. Emerson, Wm. Kenneth, J Solomonson, Peter Broyles, J. Sampson, Hertford Bros., to January 1883; L. Melton, to February 1887.

